# International Policy Approaches for Higher THC Cannabis Products: Are They Applicable to the Australian Medicinal Cannabis Framework?

**DOI:** 10.1111/dar.70203

**Published:** 2026-07-15

**Authors:** Myfanwy Graham, Dereje Assefa, Adrian Carter, Suzanne Nielsen

**Affiliations:** ^1^ Monash Addiction Research Centre, Eastern Health Clinical School, Monash University Melbourne Australia; ^2^ School of Psychological Sciences and the Turner Institute for Brain and Mental Health, Monash University Melbourne Australia; ^3^ Monash Bioethics Centre, School of Philosophy, History and International Studies, Monash University Melbourne Australia

**Keywords:** delta‐9‐tetrahydrocannabinol, high THC potency, medicinal cannabis, policy, public health

## Abstract

**Introduction:**

There have been concerted research efforts internationally to examine the health effects of higher delta‐9‐tetrahydrocannabinol (THC) concentration cannabis products and to design policies to reduce potential negative health effects of their use. This study identifies international policy approaches for regulating higher THC products relevant to the Australian medicinal cannabis program.

**Methods:**

An environmental scan of international literature and policy as part of a broader study commissioned to inform Australian policy. Methods were prospectively registered with the Open Science Framework: 10.17605/OSF.IO/HNFUT. Peer‐reviewed and grey literature on international policy approaches were identified through searches for ‘high potency THC’ and ‘policy’. The literature was then narratively synthesised.

**Results:**

We identified 76 publications and other sources that described policy approaches recommended or implemented in five jurisdictions: Canada; Germany; the Netherlands; the United States; and Uruguay. Approaches included setting a threshold for cannabis flower THC concentration at or below the biological THC limit of 30%–35% found in the plant and a risk‐tiered model for highly concentrated THC dosage forms. THC limits per dose and per package have been applied to edible products. Additional approaches included quantity limits, packaging and labelling requirements, pharmacovigilance, and education.

**Discussion and Conclusions:**

Policy approaches for higher THC concentration cannabis products could be adapted to the Australian regulatory environment to reduce potentially harmful THC exposure.

## Key Point Summary

- Exposure to higher THC concentration medicinal products may result in undesirable health effects.
- Our international policy scan identified approaches that may reduce harmful THC exposure.
- International policy approaches identified that are relevant to therapeutic use include: setting THC concentration thresholds at or below the biological THC limit of the plant; applying a risk‐tiered approach to highly concentrated THC products; supply (i.e., dispensing) boundaries; strategies to reduce appeal to youth and children; and public health education about the risks of higher THC products.
- The transferability, evidence and potential unintended consequences of implementing international policy approaches within the Australian medicinal cannabis framework are examined.

## 1 Introduction

The 2024 National Academies of Sciences, Engineering, and Medicine Cannabis Policy Landscape report identified high delta‐9‐tetrahydrocannabinol (THC) concentration products as a leading public health and safety concern in legal cannabis markets [1]. Various policy approaches have been proposed or implemented to limit the use of higher THC concentration products and reduce potential public health harms. These harms include acute presentations such as psychosis, cannabis hyperemesis syndrome, accidental or inadvertent overconsumption, and chronic harms such as cannabis use disorder [2]. These policy approaches align with the intentions of the Australian medicinal cannabis program to provide safe patient access to cannabis products suitable for medicinal use that adhere to quality standards.

Definitions of THC potency vary in published literature and in different jurisdictions [1, 3]. Recent publications have classified non‐medical THC concentration categories as ‘low potency’ (1%–9.9%), ‘mid potency’ (10%–19.9%), ‘high potency’ (20%–35%) and ‘very high potency’ (> 50%) [4]. Some THC concentrations are much higher than the biological ceiling found naturally in cannabis (~30%–35%) [5]. In medical contexts, the threshold for what constitutes a higher THC product differs substantially. The highest THC concentration in products consistent with the Australian medicinal cannabis framework, as examined in randomised controlled trials, is 22% [6]. Product potencies considered higher THC in medical contexts are often substantially lower (e.g., < 10% THC). Lower THC concentrations and doses for analgesia may result in fewer adverse events and lower rates of treatment discontinuation [7].

Increases in average THC concentrations have occurred over time in many jurisdictions [8, 9]. For example, since the legalisation of non‐medical cannabis and first legal retail sales in Colorado in 2014, the THC content of legal non‐medical flower has increased from 14% in 2014 to 19% in 2020. Concentrate product THC concentration increased from 46% to 68% [3]. Similar increases have been observed in the Australian medicinal cannabis program [10]. In contrast, in a study of THC content in illicit cannabis from Australia, only a 1% increase was estimated over the past decade. The average total THC content of 12% [11], is far lower than the THC concentrations available via prescription of ≥ 88% THC.

In Australia, only two out of more than 1000 medicinal cannabis products have undergone pre‐market Therapeutic Goods Administration (TGA) evaluation for quality, safety and efficacy [12]. Most medicinal cannabis products in Australia remain unregistered, removing a level of safety usually present in therapeutic goods. Unregistered medicinal cannabis products are available via a Special Access Scheme Category B (per patient basis) and Authorised Prescriber Scheme (classes of patients) with the intention to provide access for treatment‐resistant conditions. Medicinal cannabis products are grouped into categories based on proportional cannabidiol (CBD) content compared to total cannabinoid content under the Australian medicinal cannabis framework. The highest THC concentration products are in ‘Category 5’,^1^ defined by the TGA as THC/other cannabinoid (CBD < 2%). There is no upper limit for THC concentration. Category 5 products for inhalation, such as dried herb and concentrated extract, range from 13.2% to >88% THC. Oral liquids in this category range from 20 to 100 mg/mL THC. Other categories include Category 1 (CBD ≥ 98%), Category 2 (CBD dominant ≥ 60% and < 98%), Category 3 (balanced CBD ≥ 40% and < 60%) and Category 4 (THC/other cannabinoid dominant CBD ≥ 2% and < 40%) [10]. There is also a wide variety of unregistered medicinal cannabis product dosage forms. These include oral, inhaled and topical products [6]. Some forms, such as ‘bubble hash,’ ‘dabs’ and ‘shatter’ concentrates in the highest THC medicinal cannabis category in Australia, are similar in THC concentration to products found in North America [1].

The TGA also regulates medical devices in Australia. Only a limited number of dried herb vaporisers (e.g., MightyMedic, Volcano) and a metered dose inhaler (e.g., Syqe) are registered for administering medicinal cannabis [13]. These devices are different from cannabinoid‐containing liquid e‐cigarette devices. Unlike nicotine e‐cigarette devices, they are currently exempt from several regulatory requirements designed to protect consumers. In Australia, smoking medicinal cannabis is not recommended due to the known harmful impacts on health. Inhaled modes of administration provide a more rapid onset of effect for acute symptom relief. However, they can also negatively affect respiratory health and result in variable doses and pharmacokinetics [14]. Although there is considerable consumer demand for inhaled medicinal cannabis products, Australian observational research is limited. There is limited data on dispensed products, patient administration practices, long term health outcomes and adverse event reports for inhaled administration [15].

The medicinal cannabis prescription and dispensing model in Australia relies on health professional oversight to ensure clinically appropriate and safe patient access to products that meet quality standards. During the early implementation of Australia's medicinal cannabis program, barriers to patient access were evident [16]. The pendulum has now swung in the opposite direction, with easier patient access, the rise of online telehealth clinics, and direct‐to‐door delivery [17]. As clinical care models changed, prescription applications moved from CBD and balanced CBD/THC oral liquids toward products with the highest THC concentrations. Between 2024 and 2025, more than half of Special Access Scheme Category B prescription application approvals were for the highest THC concentration (Category 5) medicinal cannabis products [18]. These trends appear to be somewhat driven by increased prescription approval applications for inhaled Category 5 products for chronic pain and mental health indications, such as anxiety, in young males. Concerns about inappropriate supply models and rising harms have prompted health professional stakeholder groups to advocate for removing Category 5 products, adopting a risk‐tiered approach or limiting their prescription to specialists [19, 20, 21, 22].

Medical oversight and regulated product access in Australia have not always protected against harms from higher THC products. As prescribing patterns have changed, emerging evidence of harm has appeared in some peer‐reviewed studies. For example, an analysis of voluntary adverse event reports to the TGA from 6 July 2022 to 31 May 2025, found that half of all case reports (*n* = 332/614; 54.1%) were now related to the higher THC category (Category 5) of medicinal cannabis products. Psychiatric disorders were the most common adverse events for these products. Other reported events included cannabinoid hyperemesis syndrome and a case of accidental paediatric exposure to an edible product [15]. Reports of psychosis have also been noted in a retrospective audit of patients with medicinal cannabis prescriptions referred to an early psychosis service [23].

Similar trends have been observed in cannabis‐related Poisons Centre, ambulance, emergency department and hospitalisation data. However, these data do not necessarily differentiate between legal and non‐legal sources of cannabis [24]. There is also limited information about the diversion of prescribed medicinal cannabis to illicit markets and its potential impact on reported harms.

In light of these concerns, the Australian medicines regulator, the TGA, is currently considering regulatory reform of the medicinal cannabis program [25]. Aims of the regulatory reform noted in public stakeholder consultation documentation are:

that products being supplied are of appropriate quality, there is confidence in the level of safety, efficacy and performance, and further evidence is being generated to support legitimate use as a therapeutic good.

The initial stakeholder consultation process yielded more than 750 submissions, with further consultation planned.

There are rising safety concerns about potentially harmful levels of THC exposure from prescribed medicinal cannabis in Australia [17, 20, 23]. An overview of policy options is necessary and timely. This environmental scan identifies international approaches to higher THC concentration products and examines their applicability to higher THC cannabis products in the Australian medicinal cannabis program.

## 2 Methods

An environmental scan approach [26] was selected to support the timely provision of a range of policy options for the Australian medicinal cannabis regulatory framework. This policy sub‐study was conducted as part of a larger study prospectively registered with the Open Science Framework (10.17605/OSF.IO/HNFUT).

### 2.1 Search Strategy: Academic and Grey Literature

A comprehensive literature search was conducted across six databases (Medline, EMBASE, EMCARE, CINAHL, Scopus and the Cochrane Central Register of Controlled Trials) covering 1 January 2014 to 30 July 2024, as part of a rapid review with no country limits. During the rapid review, articles were tagged as policy relevant during title and abstract screening. For grey literature Google searches, the first 100 links were screened to identify national and international reports and government webpage content. Key search terms included ‘high potency THC’ and ‘policy’. Additional targeted searches of databases and grey literature were conducted to identify policy‐related publications and effectiveness evidence through 1 April 2026.

### 2.2 Resource Selection

Eligible literature included peer‐reviewed research, reports, and government webpage content describing policy approaches to higher THC concentration products. Resources about policy approaches for higher THC concentration products in medical or non‐medical (recreational) markets were included, where the approach could also be applied to the Australian context.

### 2.3 Data Synthesis

Relevant policy approaches were identified and summarised, with common features grouped thematically to support a narrative synthesis. The potential applicability and feasibility of adopting these approaches within the Australian medicinal cannabis program were then examined.

## 3 Results

Seventy‐six eligible resources between 2017 and 2026 were identified in the environmental scan. Fifty‐seven resources with information relevant to one or more policy dimensions were included in the narrative synthesis, with the balance included throughout the manuscript. These focused on policy approaches in Canada, Germany, the Netherlands, the United States and Uruguay. Five main policy approaches were identified as potentially applicable to the Australian medicinal cannabis regulatory framework. These approaches include: (i) setting thresholds for THC concentration and dosage forms; (ii) establishing THC supply (i.e., dispensing) limits; (iii) implementing strategies to reduce potential harms in paediatric populations, youth and young people; (iv) developing a comprehensive pharmacovigilance surveillance program; and (v) promoting public health‐focused education.

### 3.1 Jurisdictional Policy Approaches

Overarching policy aims in jurisdictional cannabis frameworks include reducing risks to public health and decreasing reliance on illicit markets, which can expose individuals to criminal activity (see Box 1) [27, 28]. Many policy approaches set product quality standards to minimise safety risks from adulteration or contamination. These quality standards are a core component of public health focused initiatives. Policy aims provide the foundation for different approaches adopted in each jurisdiction.

BOX 1 **Aims of the Canadian *Cannabis Act* and Regulations.**

- Public health and safety‐oriented elements aim to reduce the risk of:
  ○ Overconsumption
  ○ Unintentional consumption
  ○ Appeal to youth and young people
  ○ Food poisoning
  ○ Potential toxicity, addiction and harmful unintended effects, including adverse reactions
  ○ Diversion to the illicit market.
- Allow access for medical purposes.

International policy models encompass a range of legal and economic components. In the Netherlands, the medical program is in a transition phase following a longstanding state monopoly approach, with a tolerated non‐medical market involving licensed coffee shops also present [29, 30]. Uruguay operates a state controlled market for both non‐medical and medical cannabis, which includes provisions for home cultivation, social clubs and pharmacy‐based dispensing [5]. In Canada, both medical and non‐medical cannabis use are legalised at the federal level, with provincial and territorial differences in retail sales and home cultivation allowances for non‐medical use [31]. US state‐based commercialised models generate revenue through taxation of retail‐based sales, and most states integrate social justice and equity initiatives, though specific approaches differ [1]. Germany's more recent non‐medical framework incorporates retail sales, home cultivation and social clubs [32].

Jurisdictional medical cannabis policy frameworks differ in the degree of involvement of health professionals. In North America, authorisation or recommendation by a medical provider is required, whereas in the Netherlands and Germany, prescription‐based models are used [33, 34, 35]. Pharmacist involvement in retail sale dispensaries also varies between US states. This differs from pharmacy‐based dispensing in Uruguay, Germany and the Netherlands [29, 36, 37, 38].

### 3.2 THC Concentration Thresholds and Dosage Form Considerations

The availability of higher THC products and highly concentrated dosage forms differs between US states, Canada, the Netherlands, Uruguay and Germany. Twenty‐eight publications discussed THC concentration limits as a strategy to reduce potentially harmful THC exposure in medical and/or non‐medical frameworks [32, 39, 40, 41, 42, 43, 44, 45, 46, 47, 48, 49]. THC concentration limits differed by country and dosage form, and there is no universal consensus on the ideal THC concentration threshold [50]. Whether these limits are written into law or set by regulatory agencies via delegated powers also varies by jurisdiction.

#### 3.2.1 Flower and Concentrates

In the US, THC‐infused products are available, where the THC content of a flower product is increased by adding THC [39]. Conversely, in Québec, Canada, dried herb cannabis sold via the Société québécoise du cannabis can only contain THC concentrations that occur naturally in the plant [51]. The biological threshold of THC in cannabis plants is approximately 30%–35%. A Californian high potency think tank, using a Delphi consensus approach, recommended limiting the THC concentration of flower products to 25% [39]. There is considerable jurisdictional variance in medical and non‐medical THC concentration thresholds (see Table 1).

**TABLE 1:** Jurisdictional non‐medical and medical cannabis THC concentration threshold examples.

	Legislation (± Act and Regulations)	Dried flower and Concentrates
United States^a^		
*Vermont* [40, 41, 42, 52]	S.54 (Act 164) An Act relating to the regulation of cannabis	***Non‐medical***
		30% THC concentration limit dried flower. 60% THC concentration limit for concentrates.
		***Medical***
		No THC concentration limits.
*Connecticut* [40, 42, 53]	Public Act 21–1, An Act Concerning Responsible and Equitable Regulation of Adult‐Use Cannabis. Subsequent amendments include Public Act 22–103, An Act Concerning Cannabis and An Act Concerning Cannabis Regulation.	***Non‐medical***
		30%–35% THC concentration limit dried flower. 60%–70% THC concentration limit for concentrates. No THC concentration limit for prefilled cartridges.
	House Bill 5389 and House Bill 5450	***Medical***
		No THC concentration limits.
*Mississippi* [54, 55]	Mississippi Medical Cannabis Act (Senate Bill No. 2095). Subsequent legislation has been passed HB 1158.	***Non‐medical***
		Not permitted.
	Mississippi Medical Cannabis Program Regulations	***Medical***
		30% THC concentration limit for cannabis flower. 60% THC concentration limit for concentrates.
*Montana* [43, 56]	Initiative 190 Montana Marijuana Regulation and Taxation Act Initiative 148 Montana Medical Marijuana Act SB 423 Initiative 182	***Non‐medical***
		35% THC concentration limit for flower.
		***Medical***
		No THC concentration limits.
Canada		
*Québec* [44, 51]	Québec The Cannabis Regulation Act applies to non‐medical cannabis. For medical cannabis only Chapter IV on the restrictive use of cannabis applies.	***Non‐medical***
		THC concentration of cannabis sold via the SQDC is 30% weight per weight.
Germany [32, 57, 58]		
	Cannabis Act Cannabisgesetz (CanG).	***Non‐medical***
		Age‐based THC concentration limits of 10% are in place for 18–20‐year‐olds due to neurodevelopmental considerations. Production of THC extract and concentrate products is not permitted.
	Medical Cannabis Act Medizinal‐Cannabisgesetz (MedCanG)	***Medical***
		Medical doctor prescription of Sativex, Epidyolex, nabilone and dronabinol and a range of dosage forms dispensed in pharmacies (e.g., dried cannabis flower and extemporaneously compounded extracts). No upper THC concentration limits.
Uruguay [5, 37, 45, 59]		
	Law No. 19.172	***Non‐medical***
		Pharmacy supplied cannabis flower in Uruguay is currently 20% THC, although there is no upper limit specified in law or regulation. Additional access pathways include self‐cultivation and cannabis social clubs. Non‐flower products are not permitted.
	Law No. 19.847 (2019) that relates to magistral preparations	***Medical***
		The upper THC concentration of pharmacy supplied cannabis flower is the same for medical and non‐medical use. Licensed medical professionals can prescribe a broader range of dosage forms (e.g., flower, oil, resin) that are dispensed in pharmacies, including magistral preparations extemporaneously compounded by pharmacists.
The Netherlands [30, 46, 60]		
	Instructions of the Opium Act	***Non‐medical***
		Illegal, although there is a toleration policy (i.e., ‘coffee shops’). Average THC concentration for cannabis is 13.8% and for hashish is 26.3%.
	Medicines Act	***Medical***
		The higher end of THC concentration available in medicinal cannabis products is ~22%.

Abbreviations: SQDC, Société québécoise du cannabis; THC, delta‐9‐tetrahydrocannabinol.

^a^ Federally illegal, Schedule I—*Controlled Substances Act* and state‐specific details as per February 2026.

#### 3.2.2 Edible Dosage Forms

THC concentration limits per ‘edible’ dose unit (e.g., ‘serving size’) and total quantity per package have been implemented in some US states and Canada [47, 48]. Connecticut and Vermont set a THC limit of 5 mg, and Massachusetts implemented a 5.5 mg limit per edible dose unit for non‐medical use [49]. A 10 mg THC limit per dose unit is in place in other US states for non‐medical use. In Canada, there is a maximum of 10 mg THC per package for edible dosage forms for both medical and non‐medical use [48]. This is substantially lower than the total THC per package in some US states for non‐medical edible products, which range from 50 to 110 mg THC [49]. Colorado is an example of where escalating accidental ingestion and overconsumption of edibles led to the introduction of a limit of 10 mg THC per serving and 100 mg per package, along with packaging and labelling measures. Additional provisions related to edible dosage forms vary by state and include purchase and possession limits [61, 62].

### 3.3 Purchase, Dispensing and Possession Limits

Moderating the quantity of cannabis or THC sold, dispensed, and possessed is a mechanism to reduce higher risk consumption patterns, accidental ingestion, and diversion to the illicit market. Three publications discussed weight‐based limits in Canada, Germany, and Uruguay [32, 48, 63]. Four publications discussed weight‐based limits and/or provisions in US state‐based medical and non‐medical programs (see Box 2) [61, 62, 64, 65]. Provisions in medical contexts vary and may be equivalent to or more individualised than those in non‐medical contexts [58, 61, 62]. Nevertheless, weight‐based limits without accompanying THC concentration limits can have unintended consequences. These include the potential to supply large numbers of doses or inadvertently encourage the use of more concentrated products [61, 62, 65].

BOX 2 **Examples of Mechanisms to Moderate Quantity.**

- In medical models, dose and overall quantity dispensed may be individualised on a per‐patient basis by their treating physician or a pharmacist.
- Sale or dispensing limits may involve one or a combination of the following approaches:
  ○ Per transaction or time interval based (e.g., daily, weekly).
  ○ Product weight‐based.
  ○ THC concentration‐based.
  These approaches may also include a minimum age or age‐based limits for non‐medical programs. In comparison, medical programs may include provisions for paediatric populations and their caregivers.
- Possession limits
  ○ Medical exemptions or additional clinical justification are required for larger quantities.
  ○ Weight‐based limits in non‐medical contexts.

### 3.4 Policy Approaches to Protect Paediatric Populations, Youth and Young People

Cannabis packaging and product characteristics that may appeal to children and younger populations include illustrations, references to foods or flavours, specific product flavours, cartoon characters, bubble‐like font and gummy bear forms [66, 67, 68, 69, 70]. Edibles are significantly more appealing to younger populations than older adults [67]. Inadvertent paediatric poisonings have been reported with edible products internationally [71, 72, 73, 74]. Four age‐based policy approaches have been proposed or implemented in three countries with non‐medical markets, aiming to reduce unintentional ingestion by paediatric populations and the appeal of higher THC concentration products to young people. These approaches include: (i) plain packaging and health warnings; (ii) product characteristic limits (e.g., flavours, dosage form); (iii) THC concentration limits; and (iv) supply limits [32, 39, 48, 66, 67, 68, 69, 70, 75, 76, 77, 78].

A Health Canada policy statement details product, packaging, labelling, and promotional factors that may increase appeal to young persons [78]. Policy measures to reduce unintentional paediatric ingestion and youth appeal have been implemented for both medical and non‐medical cannabis products in Canada. In Québec, Canada, edible flavours are limited to those that do not appeal to youth, such as beets and cauliflower [75]. In Nova Scotia and Newfoundland, flavour additives are not permitted in electronic cigarette cannabis forms [75]. Other safety measures in Canada include child‐resistant packaging for most dosage forms as well as plain product packaging with warning labels [48].

Additional policies regarding the addition of excipients (e.g., flavours) and packaging have been proposed in the US to reduce the appeal of cannabis products to young people. Recommendations include limiting the addition of both natural and synthetic flavours to inhaled products. In Watsonville and Contra Costa counties in California, flavoured inhaled cannabis products are not permitted, regardless of whether they are for medical or non‐medical use. Further proposals suggest that label language and imagery that are suggestive of flavours other than cannabis should be avoided. A Californian report on policy approaches for higher THC concentration products also recommended the consideration of graphic health warnings on packaging, similar to tobacco products [39].

### 3.5 Comprehensive Pharmacovigilance Strategies

Five publications described pharmacovigilance strategies designed to collect comprehensive data on the safety profile of THC‐containing cannabis products. Prescription drug monitoring programs (PDMP) collect patient‐level data, which can help identify individuals at risk. This information supports clinical decision‐making by both prescribers and pharmacists. Several US states (e.g., New York, Connecticut, Virginia, North Dakota, Arizona and Ohio) have added medical cannabis to their PDMP. One study reported a statistically significant decrease in contraindicated medication fills when cannabis was added to a state PDMP [79]. Data capture via national adverse event data repositories has improved understanding of adverse events and cannabinoid‐drug interactions [80, 81, 82, 83].

### 3.6 Education

Consumer focused public health education has been identified as a way to reduce potential public health harms related to higher THC cannabis products [39]. In 2024, the Colorado School of Public Health launched a public health initiative called ‘the tea on THC’. This campaign communicates the potential harms of high THC concentration products, especially for vulnerable groups such as youth and individuals who are pregnant or lactating [65, 84]. Health Canada has created school‐based educational resources for teenagers. These materials aim to raise awareness of how cannabis use may impact mental health and brain function [85]. Canada's lower risk use guidelines for non‐medical cannabis recommend avoiding high THC concentration products. The guidelines also provide advice on safer use practices [86].

There are limited studies on the effectiveness of health professional or patient education initiatives, and none are specific to higher strength THC products. North American studies of the effectiveness of nurse practitioner and pharmacist student education reported improved knowledge and preparedness to engage with patients about cannabis [87, 88]. A retrospective survey based analysis examined a mandatory, pharmacist‐led medical cannabis education session for patients with chronic pain. Following the session, half of the patients using medical cannabis indicated they would select a low THC product. Participants were also more likely to be aware of potential adverse effects after receiving education [89]. A retrospective chart review examined a pharmacist‐led cannabis consultation service that offered education and counselling to patients with cancer. Most patients (75%) chose a low THC, high CBD oral oil (2:50). Collectively, these studies suggest that pharmacist‐led education initiatives may encourage patients to select low THC products [90].

Public awareness of the potential risks of medicinal cannabis is essential as rates of use increase. Systematic reviews indicate that health warnings on cannabis product packaging may increase consumer knowledge and reduce the appeal of these products when potential harms outweigh benefits [91]. The effectiveness of mandated health warnings on cannabis products has also been examined. Canadian health warnings provide information about the risks of higher THC products and their effects on mental health and cognition. The warnings also address health harms linked to smoking and give specific safety guidance for vulnerable groups, including pregnant or lactating individuals, adolescents and young adults [92]. Some studies have shown that pictorial health warnings are more effective than text‐based warnings at reducing the appeal of cannabis products, although findings on their effectiveness have been mixed [91]. In Germany, product packaging requirements include cannabinoid composition information, health warnings and neutral appearance [32].

Qualitative studies from the US and Canada found that consumers often interpret cannabis health warnings differently than intended. Health Canada used focus groups to test cannabis health warning messages. Participants preferred warnings with specific numerical thresholds over general references to ‘higher strength products’ [93]. Consumers have expressed confusion about statements on product regulatory oversight and warnings that conflict with product instructions. There is also a mismatch between consumer perceptions of safety, personal experiences and the content of health warnings. Consumer indifference to health warnings when making purchasing decisions has also been reported. In experimental studies that examined believability and effectiveness, health messages about addiction have been reported to be the least effective [91].

## 4 Discussion

Five broad policy approaches were identified that could be applied or adapted as risk mitigation strategies for higher THC medicinal cannabis products in Australia. These policy options could be considered individually or in combination to achieve the goal of reducing potentially harmful THC exposure.

### 4.1 Inhaled Dried Herb and Concentrated Extract THC Concentration Thresholds

Different concentration thresholds for THC were identified in US states and for non‐medical use in Germany [32, 39, 40, 41, 43, 53, 54]. In Uruguay, the Netherlands, and Québec, Canada, product availability shapes the upper THC concentration that is available [5, 46, 51]. There is no upper THC concentration threshold in place in Australia. Most Australian Category 5 products fall between 13.2% and 88% THC. Some products categorised as ‘herb, dried’ have THC concentrations as high as 60%. These levels exceed the biological ceiling limit of the plant, which is approximately 30%–35% THC [5, 6]. In the Australian medicinal cannabis market, there are over 1000 different products [12]. Available dosage forms are diverse and include oral liquid, dried herb, oil, solution, capsule, pastille, spray, wafer, pressurised inhalation, topical, lozenge, concentrated extract, dry extract, tincture, chewable tablet, gel, pessary, tablet, suppository and chewing gum [18].

Australian medicinal cannabis regulators could consider a variety of policy options to limit exposure to potentially harmful concentrations of THC. For inhaled products, a THC concentration threshold of approximately 22% could be adopted. This is based on the upper limit used in randomised controlled trials evaluating clinical applications [6]. This approach would need to be combined with regulatory controls on excipients, such as diluents, used to stay within THC thresholds. A risk tiered approach would involve stratifying products by risk, including low, medium, high and very high risk, and applying policy measures accordingly. This risk tiered approach could be applied to highly concentrated inhaled dosage forms (e.g., 75%–88% THC), with either removal from the medical market or additional requirements for prescription. Removing solid THC concentrates from the market may have unintended consequences. These include consumers seeking unregulated sources, increased use of adulterated or contaminated unregulated products, and risk of explosions and fires with illicit manufacture [94, 95]. Another option would restrict the addition of concentrates to dried herb that increases THC concentrations above the biological ceiling of THC naturally found in the plant, ~30%–35%. Limits specified in legislation may be more likely to remain in place and less susceptible to fluctuation. Providing adequate prospective public notice of THC concentration thresholds for inhaled products would give patients the opportunity to transition to other dosage forms. This would allow for dose titration and help reduce the risk of withdrawal.

THC concentration limits do not prevent individuals from consuming higher overall doses, as individuals can simply use larger quantities of lower strength products. Evidence is mixed on whether people reduce the amount they use when consuming higher THC products [96]. Studies indicate that reducing the quantity used with higher THC products may not fully offset the effects of the higher concentration. Moreover, some consumers choose not to reduce their intake at all [96, 97, 98]. When assessing potential harms, it is important to consider not only THC concentration but also the frequency, quantity and duration of cannabis use [99].

A common concern with imposing THC concentration limits is that consumers may turn to the illicit market, where product composition and quality are uncertain. Patients with complex chronic health conditions and those who are immunocompromised may be especially vulnerable to health risks from adulterated or contaminated products [100]. Therefore, any implementation of THC thresholds should include public education about the differences between regulated and unregulated products to address these potential risks. The EVALI outbreak in the US is a frequently noted example of the harmful impacts of unregulated product use [95]. However, Australian medicinal cannabis guidelines address concerns that go beyond adulterants and unknown contents. They highlight risks associated with inhaled products and their impact on respiratory health and clearly recommend oral products instead [14]. A comprehensive review of regulatory models for e‐cigarette products, especially those intended for non‐medical use, is outside of the scope of this paper. Proposed regulatory reforms should strive to balance interim patient access with the transition to registered and approved products while reducing potential harm.

### 4.2 Pastille ‘Edible’ THC Concentration Limits and Packaging

Unlike the North American examples discussed, Australia has not set THC concentration thresholds per dose and package for medicinal cannabis pastille products [39, 47, 48, 49]. In Australia, Special Access Scheme Category B prescription approvals for Category 5 pastille medicinal cannabis dosage forms (commonly referred to as ‘edibles’ or ‘gummies’) rose from 137 in 2023 to 2722 in 2024, and then to 8253 in 2025 [18]. It will likely be some time before any reported effects of this are published. Category 5 product THC doses per pastille range from 5 to 30 mg. Total THC content per package ranges from 300 to 1200 mg [10]. Ensuring the availability of products containing lower THC concentrations (such as 2.5 mg or less) would better align with medical use requirements. These strengths also enable lower starting doses and gradual dose adjustments.

The risk of inadvertent consumption of ‘edible’ products and severe poisoning in children could be reduced by several strategies. Regulatory limits could be established for THC permitted in a single dose unit. Single dose unit packaging, similar to blister packs used for other medicines, could further enhance safety. Another strategy is to restrict or eliminate dosage forms and organoleptic properties that are attractive to children, such as confectionery‐like products. Requiring child‐resistant packaging provides an important layer of protection against unintentional access by children. Additionally, educating parents and caregivers on safe storage practices can help ensure these products are kept out of children's reach [72, 101, 102]. Until further data on the impact of increased edibles prescription approvals in Australia becomes available, a proactive risk‐mitigation approach informed by international best practices could prevent unnecessary harm.

The delayed onset of effects with edible cannabis products can result in repeated dosing before the maximum effect of the first dose. This may lead to accidentally consuming too high a dose and experiencing undesirable effects, especially in individuals who are cannabis naïve [103]. Providing patients with information on what constitutes a therapeutic single dose, standard dose intervals, and maximum recommended doses can help avoid undesirable effects [70, 104]. International limits on total package THC quantity (e.g., 10 mg, 100 mg) would need to be adapted to a prescription‐based model where daily dosing may be prescribed for chronic health conditions. It is standard practice to dispense a month's supply, typically at 30‐day intervals, and higher cumulative doses may be required as patients develop tolerance to THC.

Supply limit mechanisms such as authority prescriptions and staged supply, which are currently used for some Pharmaceutical Benefits Scheme (PBS) subsidised prescriptions, could be considered. Since most Australian medicinal cannabis products are not included in the PBS, these approaches would need to be modified accordingly. Dispensing smaller quantities or extemporaneously manufactured products may require opening manufacturer packaging. This process could result in repackaging products in containers lacking safety features designed to prevent accidental paediatric consumption. This highlights the need for pack sizes that reflect quantities required for therapeutic use. Regulators could also consider setting dose unit THC concentration limits of edible products to 5–10 mg per dose. This could be paired with dispensing quantity limits and intervals aligned with standard oral dosing and maximum daily THC limits.

### 4.3 Prescribing and Dispensing Limits

In the Australian medicinal cannabis program, prescriptions for THC containing products must specify both the total quantity to be dispensed and the dispensing interval. Health professional guidance specifies that prescribing and dispensing excessive quantities should be avoided [17]. Prescribed medicines in Australia are typically dispensed in 30‐ or 60‐day supplies. Similar to approaches with opioids, upper thresholds for overall THC quantity and repeat intervals could be considered. One risk‐based approach, as implemented in Western Australia, requires prescribers to provide additional justification and obtain authorisation for daily oral THC doses exceeding 40 mg or inhaled THC doses above 300 mg per day [105].

Current medicinal cannabis dose guidance is based on ‘starting low and going slow’. A THC standard unit has also been proposed [106]. This concept would require adaptation for the lower doses used in therapeutic settings, and further pharmacokinetic research is needed to support its application across different dosage forms. Parallels can be drawn with opioids, where harms were identified with ongoing escalation to very high doses. Best practice includes providing clear guidance on maximum daily doses and issuing warnings against continuous dose increases. For opioids, daily oral morphine equivalent thresholds of 50–100 mg and above 100 mg have been established and can be flagged in prescription monitoring systems. In contrast, additional research is necessary for medicinal cannabis to establish bioequivalence metrics and to determine what constitutes a ‘high’ or ‘very high’ prescribed THC dose across various dosage forms and indications. Defining these dose ranges and thresholds would assist recognition of when a prescribed dose exceeds the limits supported by current evidence for therapeutic use.

Prescribing and dispensing approaches based on THC thresholds should account for the need to individualise cannabinoid doses. They must also consider situations where larger quantities may be required. For example, tolerance with daily or long‐term use for chronic conditions and palliative care. Prescribing and dispensing limits can reduce overall doses, which may benefit patients new to medicinal cannabis. Although caution is warranted, as lessons learned from the unintended consequences of opioid dose limits for patients already on high doses may also apply to medicinal cannabis. Further research is needed to determine whether similar restrictions are appropriate for medicinal cannabis [107].

### 4.4 Policy Approaches to Reduce Potential Harm in Paediatric Populations and Young People

A range of policies have been proposed or are in place to reduce potential harms from cannabis in paediatric and young populations [32, 39, 48, 67, 75, 76]. Measures that have been applied internationally could also help prevent accidental poisoning in children and make higher THC products less appealing to young people.

Enhancing risk mitigation measures in the Australian medicinal cannabis regulatory framework could reduce potential harms for paediatric and young populations. In Australia, a medical specialist's letter is required for the prescription of a medicinal cannabis product containing THC to an individual less than 18 years old [108]. While there are existing packaging and labelling requirements for prescribed products in Australia, there are also important exemptions to these requirements [109]. Most products have child‐resistant packaging, except flower based and e‐cigarette products, which are exempt from this requirement. Additional precautions include warning labels advising patients to keep products out of reach of children [110]. Unlike most other registered medicines, not all unregistered medicinal cannabis products available in Australia apply plain packaging principles. TGA standards for nicotine e‐cigarettes on permitted flavours and excipients, plain packaging, product naming, labelling and design [111] could be applied to medicinal cannabis e‐cigarettes (i.e., ‘vapes’) and other dosage forms. Applying these standards could help reduce youth appeal if medicinal cannabis products are diverted or accidentally accessed.

Advertising restrictions are already in place in Australia. When compliance breaches are identified, the TGA has imposed fines [112]. Previous audits have reviewed composition requirements [113, 114], and medicinal cannabis remains a priority focus for TGA compliance activities. Additional resources to strengthen auditing of product characteristics, quality standards, devices, packaging and labelling would be timely.

### 4.5 Comprehensive Pharmacovigilance Surveillance Program

As in some US states, Australia has included THC‐containing medicinal cannabis products in real time prescription monitoring (RTPM) systems (e.g., SafeScript) and My Health Record. Most THC‐containing medicinal cannabis products are private prescriptions that must be individually recorded, rather than selected from a standard list in dispensing software. As a result, product details are often entered inconsistently. This can lead to inconsistencies in data capture. Applying Australian Medicinal Terminology to all medicinal cannabis products could improve how data is captured, making it easier to identify risky patterns of use or misuse.

Unintended consequences of RTPM systems have been reported, such as refusal of supply for patients with legitimate clinical needs, inadequate pain management and stigma [115]. In Australia, interstate prescribing and dispensing of medicinal cannabis adds further logistical difficulties. Health professionals must register separately with each state's RTPM system to ensure visibility. For instance, if a prescription is issued in Western Australia, a patient's General Practitioner in another state might not be aware of their medicinal cannabis use.

Additional sources of adverse event data related to medicinal cannabis [116] and translation of safety considerations to clinical practice settings have been compiled [117]. There is a requirement for mandatory prescriber reporting of unregistered medicinal cannabis product related adverse events. There is the additional opportunity to require adverse event reporting by medicinal cannabis product sponsors (i.e., companies that import or manufacture medicinal cannabis products) [118]. This would require products to be registered on the Australian Register of Therapeutic Goods, which is a notable gap for unregistered medicinal cannabis products [12].

### 4.6 Education

Evidence from the US and Canada supports the role of public health and health professional education on higher THC concentration cannabis [39, 86, 119, 120]. Australian health professionals need to maintain minimum Continuing Professional Development requirements. These education updates should reflect their scope of practice, including medicinal cannabis. The Royal Australian and New Zealand College of Psychiatrists Clinical Memorandum on the therapeutic use of medicinal cannabis products references potential harms from higher THC concentration products [121]. The Australian Health Practitioner Regulation Agency and national boards have also issued guidance on prescribing and dispensing practices for medicinal cannabis [17]. Incorporating guidance on higher THC concentration products into current health professional educational resources could help facilitate informed discussions with patients.

There is consensus amongst key stakeholder groups, including medicinal cannabis product sponsors, industry associations and professional associations, on the need for education related to medicinal cannabis [25]. Further initiatives could include the establishment of education requirements for health professionals who prescribe medicinal cannabis. Although education should be combined with other policy measures. Education on health harms with higher THC products may have less impact on reducing population level harm than policy approaches such as prohibiting advertising [122] or regulating products or professional practice. This is particularly relevant for prescribers working in profit‐driven clinical contexts that incentivise the prescribing of medicinal cannabis products. Moreover, a key priority area is generating clinical research to inform education.

Public health education initiatives for patients and carers could provide information about potential health harms from the use of higher THC products (e.g., psychosis, cannabis use disorder, cannabis withdrawal syndrome). This could incorporate practical harm reduction strategies, such as guidance on dose, frequency and route of administration, to reduce potentially harmful THC exposure. There is precedent for health warnings on medicines in Australia, including black triangle or box warnings. As a uniform approach has not yet been adopted, an industry wide harmonised approach to medicinal cannabis could be considered [123, 124]. Mandated health warnings related to higher THC concentration products could be introduced. Warnings for vulnerable populations could focus on those at risk of adverse mental health effects, people planning pregnancy, pregnant or lactating individuals, older adults and young people. Standardised label components for medicinal cannabis products could include product name, cannabinoid concentration (in milligrams and percentage) per dose unit and per package, starting dose, dose titration, maximum daily THC dose, dosage form, route of administration, safe storage, batch details and expiry date. Labels could also feature QR codes linking to detailed product information, such as precautions, drug interactions, adverse event reporting and Certificate of Analysis details.

### 4.7 Affordability and Equitable Access

Affordability and equitable access to medicinal cannabis products are key policy considerations within the Australian medicinal cannabis framework [16]. Access to most medicinal cannabis products is funded privately by patients or has partial coverage by some private health insurers. Some medicinal cannabis suppliers also have compassionate pricing options. Currently, only Epidyolex/Epidiolex (CBD 100 mg/mL) is subsidised under the PBS for Lennox–Gastaut Syndrome and Dravet Syndrome. PBS subsidisation decisions rely on high‐quality randomised controlled trial and cost‐effectiveness data. Since this data is limited for most medicinal cannabis products, PBS subsidisation is currently unlikely. As a result, the cost‐effectiveness of higher THC concentration products may unintentionally incentivise their use. Strategies considered for consumer goods in legalised non‐medical contexts, such as a THC potency tax, are less relevant to a medicine‐based regulatory framework, though they could potentially be adapted as a levy.

### 4.8 Limitations and Research Gaps

This environmental scan of international policies related to higher THC products may not identify policies for which little has been published. Cannabis policy approaches adopted or recommended in different jurisdictions are often based on policies for cannabis in non‐medical contexts or drawn from the alcohol and tobacco literature. However, their effectiveness in medical cannabis settings remains unclear [125]. A comprehensive review of lessons from alcohol and tobacco regulation that could inform cannabis policy has already been published [126]. Some studies extrapolate findings from other medicines or general cannabinoid‐related literature. An expert Delphi consensus panel evaluating state level cannabis policies in US non‐medical markets highlighted the limited scientific evidence on the efficacy of different cannabis policies [127]. Most cannabis policy studies assess outcomes related to medical cannabis legalisation overall, rather than the effects of specific policy provisions [128]. While there is a growing Australian literature on medicinal cannabis, it is largely focused on prescribing patterns and clinical indications. There is comparatively little research examining the broader context and consequences of the rapidly expanding medicinal cannabis market in Australia [15, 23, 129, 130], highlighting the need for further work in this area. Moreover, Australia is one of many countries, including the United Kingdom and Germany, examining intended and unintended outcomes of existing prescription medicinal cannabis models and considering policy approaches to balance patient access and safety [131, 132]. If such policies are implemented in Australia, conducting a rigorous evaluation would enable Australia to be a case study that could inform other jurisdictions.

## 5 Conclusion

The international policy approaches identified offer insights into managing higher THC concentration products within the Australian medicinal cannabis regulatory landscape. These include setting THC concentration thresholds for inhaled and edible products, applying a risk‐based approach to higher THC dosage forms, and establishing THC concentration‐based limits for prescribing and dispensing. Measures related to product characteristics, labelling and packaging may help reduce the appeal to youth and children. Enhanced pharmacovigilance could improve signal detection and data capture. Public health education and mandated health warnings, together with further research, could serve as risk mitigation strategies to reduce THC exposure‐related harms. These policy options aim to balance patient access to medicinal cannabis for medical use with the need to reduce potential public health harm.

## Author Contributions

**Myfanwy Graham:** conceptualisation, design, protocol development, literature searching, screening, data synthesis, visualisation, writing review and editing. **Dereje Assefa:** screening, writing review and editing. **Adrian Carter:** writing review and editing. **Suzanne Nielsen:** conceptualisation, design, protocol development, screening, writing review and editing, supervision.

## Funding

This work was commissioned by the Australian Therapeutic Goods Administration, Department of Health, Disability & Ageing. MG is the recipient of an NHMRC Postgraduate Scholarship (GNT#2030765) and a Monash Graduate Research Excellence Scholarship. SN is the recipient of an NHMRC Leadership Fellowship (GNT#2025894).

## Conflicts of Interest

M.G. is an appointed member of the Therapeutic Goods Administration's Medicinal Cannabis Expert Working Group. This article does not represent the views of the TGA or the Expert Working Group. All other authors report no conflicts of interest.

## Data Availability

The data that support the findings of this study are available from the corresponding author upon reasonable request.

## Endnotes

^1^
A small proportion of Category 5 products contain cannabinoids other than THC, such as cannabigerol.
